# Establishment of a novel axon pruning model of *Drosophila* motor neuron

**DOI:** 10.1242/bio.059535

**Published:** 2023-01-06

**Authors:** Wanyue Xu, Weiyu Kong, Ziyang Gao, Erqian Huang, Wei Xie, Su Wang, Menglong Rui

**Affiliations:** School of Life Science and Technology, the Key Laboratory of Developmental Genes and Human Disease, Southeast University, 2 Sipailou Road, Nanjing 210096, China

**Keywords:** Development, *Drosophila*, Neuroscience

## Abstract

Developmental neuronal pruning is a process by which neurons selectively remove excessive or unnecessary neurite without causing neuronal death. Importantly, this process is widely used for the refinement of neural circuits in both vertebrates and invertebrates, and may also contribute to the pathogenesis of neuropsychiatric disorders, such as autism and schizophrenia. In the peripheral nervous system (PNS), class IV dendritic arborization (da) sensory neurons of *Drosophila*, selectively remove the dendrites without losing their somas and axons, while the dendrites and axons of mushroom body (MB) γ neuron in the central nervous system (CNS) are eliminated by localized fragmentation during metamorphosis. Alternatively, dendrite pruning of ddaC neurons is usually investigated via live-cell imaging, while dissection and fixation are currently used for evaluating MB γ neuron axon pruning. Thus, an excellent model system to assess axon specific pruning directly via live-cell imaging remains elusive. Here, we report that the *Drosophila* motor neuron offers a unique advantage for studying axon pruning. Interestingly, we uncover that long-range projecting axon bundle from soma at ventral nerve cord (VNC), undergoes degeneration rather than retraction during metamorphosis. Strikingly, the pruning process of the motor axon bundle is straightforward to investigate via live imaging and it occurs approximately at 22 h after pupal formation (APF), when axon bundles are completely cleared. Consistently, the classical axon pruning regulators in the *Drosophila* MB γ neuron, including TGF-β signaling, ecdysone signaling, JNK signaling, and the ubiquitin-proteasome system are also involved in governing motor axon pruning. Finally, our findings establish an unprecedented axon pruning mode that will serve to systematically screen and identify undiscovered axon pruning regulators.

This article has an associated First Person interview with the first author of the paper.

## INTRODUCTION

During animal development, neurons generate excessive or exuberant connections at an early stage, and subsequently remodel their dendritic arbors and axon projections to wire functional circuits (Luo and O'Leary, 2005; Williams and Truman, 2005). During the metamorphosis of the holometabolous insect *Drosophila*, early-stage neurons undergo stereotyped pruning to reconstruct adult-specific neural circuits (Consoulas et al., 2000; Kanamori et al., 2015; Truman, 1990). In the central nervous system (CNS), mushroom body (MB) γ neurons eliminate the dorsal and medial axon branches as well as all dendrites and subsequently regrow the medial branches to form the adult-specific nervous system (Lee et al., 1999). Similarly, in the peripheral nervous system (PNS), a subset of dendritic arborization (da) sensory neurons, class I (ddaD/ddaE) and class IV (ddaC) neurons, remove almost all of the dendrites without losing their axons and somas, whereas class II (ddaB) and class III (ddaA/ddaF) da neurons are eliminated through apoptosis (Kuo et al., 2005; Williams and Truman, 2005). Extensive studies have suggested that ecdysone signaling is the main gatekeeper of axon/dendrite remodeling (Kirilly et al., 2009, 2011; Lee et al., 2000; Zheng et al., 2003). Furthermore, several known pathways that contribute to neurite pruning have been revealed: ubiquitin proteasomal degradation (Kuo et al., 2005; Watts et al., 2003; Wong et al., 2013), caspase activity (Kuo et al., 2006; Williams et al., 2006), microtubule disassembly (Bu et al., 2021; Herzmann et al., 2017; Lee et al., 2009) and polarity (Herzmann et al., 2018; Rui et al., 2020b; Tang et al., 2020; Wang et al., 2019), glia engulf (Awasaki et al., 2011; Awasaki and Ito, 2004; Tasdemir-Yilmaz and Freeman, 2014; Watts et al., 2004), epithelial engulf (Han et al., 2014), nitric oxide (Rabinovich et al., 2016), secretory pathway (Rui et al., 2020a; Wang et al., 2017), endocytic pathway (Issman-Zecharya and Schuldiner, 2014; Zhang et al., 2014), cell adhesion (Bornstein et al., 2015) and energy metabolism (Marzano et al., 2021). Importantly, during the past few decades, developmental neuronal pruning has been reported in various mammalian brain regions, including the hippocampus, cortex, cerebellum, and olfactory bulb (Luo and O'Leary, 2005; Riccomagno and Kolodkin, 2015; Schuldiner and Yaron, 2015). Aberrant pruning, which results in altered dendritic or axonal density in human brains, is related to neurological disorders, including autism and schizophrenia (Glantz and Lewis, 2000; Penzes et al., 2011; Tang et al., 2014). Thus, understanding the mechanisms of developmental pruning would provide important insights into the pathogenesis of human neurological disorders.

**Fig. 1.**
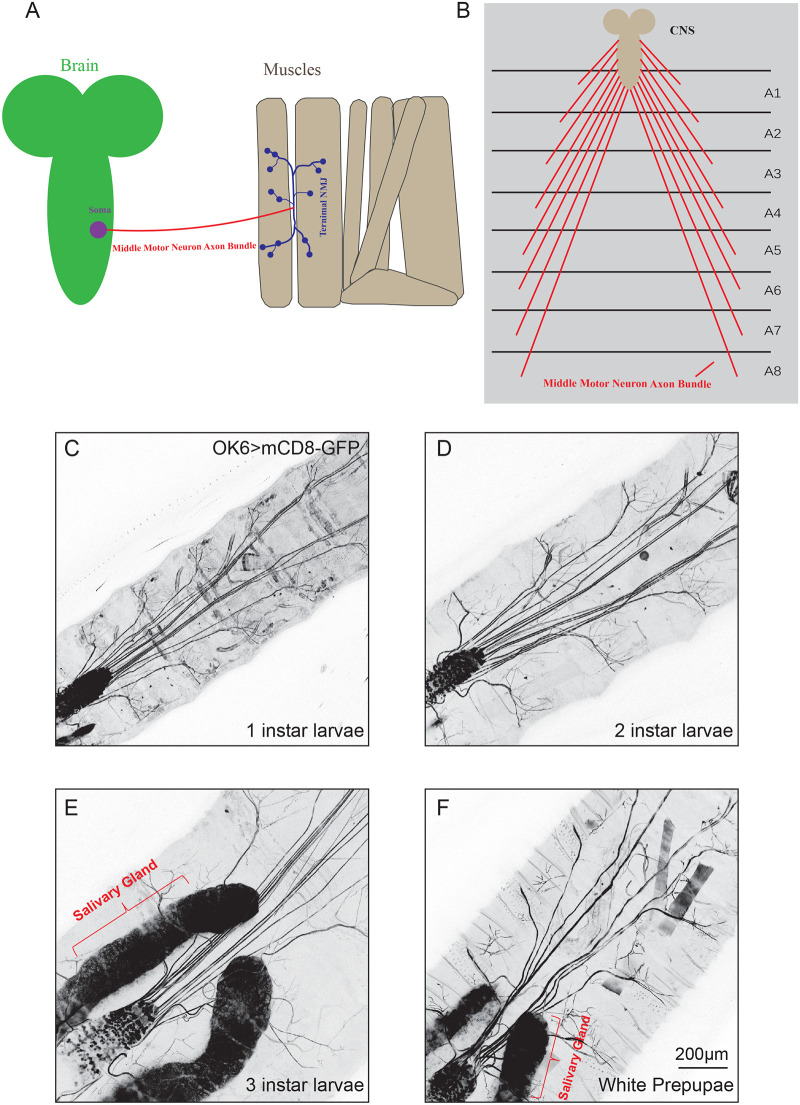
**Axon bundles of motor neuron are maintained intact from larvae to white prepupae stages.** (A) A model of axonal bundle branching in *Drosophila* motor neurons. *Drosophila* motor neuron axon bundle branching model. The middle motor neuron axon bundle (in red) extends from the VNC soma and connects to the target muscle to form a specific terminal neuromuscular junction (in blue) that innervates the muscle. (B) A schematic of the anatomy of a *Drosophila* third instar larva with its brain and VNC at the anterior end and motor neurons extending posteriorly from the VNC to eight abdominal segments on the body in a trapezoidal axonal array. (C-F) Confocal z-projections of motoneurons of first instar larvae (C), second instar larvae (D), third instar larvae (E), and white prepupae (F) with OK6-Gal4 (OK6)-driven mCD8-GFP markers of specific genotype. The mCD8-GFP is also expressed at salivary gland that is denoted with red bracket. Axons are intact and do not undergo remodeling, retraction or degeneration. Scale bar: 200 μm.

*Drosophila* larvae exhibit several forms of locomotion including peristalsis, bending, crawling, and turning. These movements have proven to be innervated by the neuromuscular system (Kohsaka et al., 2012). The motor system of *Drosophila* begins to establish during the embryonic stage and is generated in lineages derived from at least ten different neuroblasts (Landgraf et al., 1997; Schmid et al., 1999), their muscle targets consist of 11 segments including three thoracic segments, and eight abdominal segments (Keshishian et al., 1996). These motor neuron bundles stretched from ventral nerve cord (VNC) soma and navigate within the 30 muscles in each hemi-segment and finally establish specific connections with their target muscles (Arzan Zarin and Labrador, 2019). After the axonal growth cone contacts with its target muscle, postsynaptic glutamate receptors and Discs-large – the *Drosophila* ortholog of the mammalian PSD-95 postsynaptic scaffolding protein – accumulate at the contact region. The growth cone then differentiates into a presynaptic terminal (Chou et al., 2020). In addition, the ease in accessing the structure of the *Drosophila* motor axon bundle means it can be examined more conveniently (Fig. 1A,B) and therefore it might emerge as an attractive model to explore cellular and molecular mechanisms of axon-specific remodeling.

To systematically reveal new neurite pruning regulators and better understand the mechanisms of axon pruning, we monitored the elaborate developmental process from first instar larvae to 24 h after pupal formation (APF) stages and established a novel axon pruning model system using *Drosophila* motor neuron via live-cell imaging. The *Drosophila* motor neuron is a long-range projecting principal glutamatergic neurons (Chou et al., 2020; Leiserson et al., 2000; Schuster, 2006). Mechanistically, the motor neuron extends their long axon bundles from VNC to innervate muscles (Menon et al., 2013) and the stretched axon bundles are more straightforward for observing than MB γ neuron in the CNS. In particular, the developmental pruning of the motor system can be monitored via live imaging, which is limited in the MB γ neuron. Previous studies have demonstrated that the terminal component of motor neurons, *Drosophila* neuromuscular junction (NMJ), is a glutamatergic synapse (Rui et al., 2017) that has proven to be retracted during metamorphosis (Boulanger and Dura, 2015; Boulanger et al., 2012; Liu et al., 2010). Conversely, we found that the majority of axon bundles extended from VNC except NMJ via large-scale degeneration rather than retraction to reconstruct adult-specific motor neural system during the development. Thus, the *Drosophila* motor neuron provides an invaluable resource for understanding axon pruning. Furthermore, our results showed that it occurs approximately at 22 h APF in motor neuron rather 18 h APF in MB γ neuron, when almost all the axons are pruned away. Accordingly, axon pruning of the motor neuron is a highly conserved process that is controlled by several known axon pruning regulators, including TGF-β signaling, ecdysone signaling, JNK signaling and the ubiquitin-proteasome system. Collectively, we provide multiple lines of evidence demonstrating that the motor neuron of *Drosophila* can be used as an attractive model to study cellular and molecular mechanisms of axon specific pruning.

## RESULTS

### The middle axon bundles of motor neuron remain intact from larvae to white prepupae stages

To better gain insight into the elaborate architecture, we intend to investigate the overall morphology of *Drosophila* motor system via live-cell imaging during the development from first instar larvae to white prepupae stages, a period when individuals have ceased movement, everted anterior spiracles, but have not yet begun tanning of cuticle, before ecdysone signaling is activated. To this end, we constitutively overexpressed mCD8-GFP in motor neurons driven by OK6-Gal4, as a previous study revealed that OK6 expression is strongly observed in larval motor neuron axons and their neuro-muscular synapses, and OK6 also expresses in neurons of the larval brain including the larval optic lobes (Sanyal, 2009). Our imaging results suggested that almost all motor neuron axon bundles are linked with their VNC somas and muscles. Although the terminal NMJ of motor neuron is ambiguous for observation, the middle axon bundles are easily captured by confocal microscope at this resolution. Our live-cell imaging results showed that the overall axon structure was maintained intact from first instar larvae to white prepupae stages without remodeling, like retraction or degeneration (Fig. 1C-F). In order to further characterize the detailed growing status of motor neuron, we next investigated the terminal NMJ's morphology during larval to pupal stages and our immunostaining results suggested the addition of synaptic boutons and branches during larval to white prepupal growth both in NMJ4 and NMJ6/7 (Fig. 3A1-F3).

**Fig. 2.**
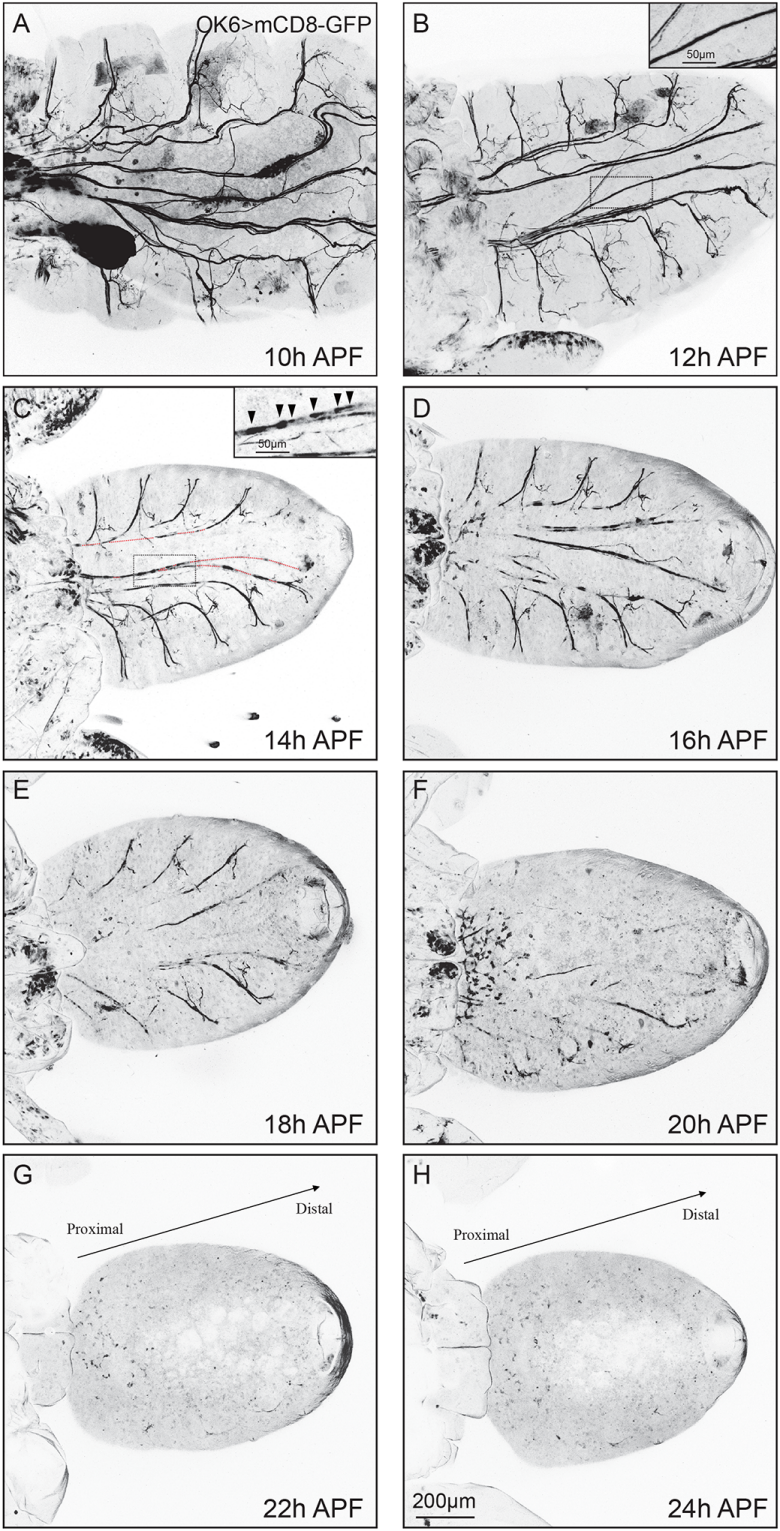
**Axon bundles of motor neuron undergo degeneration during metamorphosis.** (A-H) Representative confocal live imaging of the development of mCD8-GFP-labelled motor neurons driven with OK6-Gal4 (OK6). At 10 h APF and 12 h APF axon bundles remain intact (A,B). The zoomed image showed the continuous axonal fiber at 12 h APF (B). Discontinuous axon bundle appears in the axon at 14 h APF, indicating the start of axonal degeneration (C). The zoomed image suggested the septal membrane structure denoted by arrows could be recognized as a sign for initiating degeneration at 14 h APF (C). After 14 h APF, the degree of motor neuron pruning increased dramatically and the middle axon bundle is completely pruned at 22 h APF and 24 h APF (G,H). The proximal/distal directionality is marked (G,H). Scale bars: 200 μm (normal images), 50 μm (zoomed images).

**Fig. 3.**
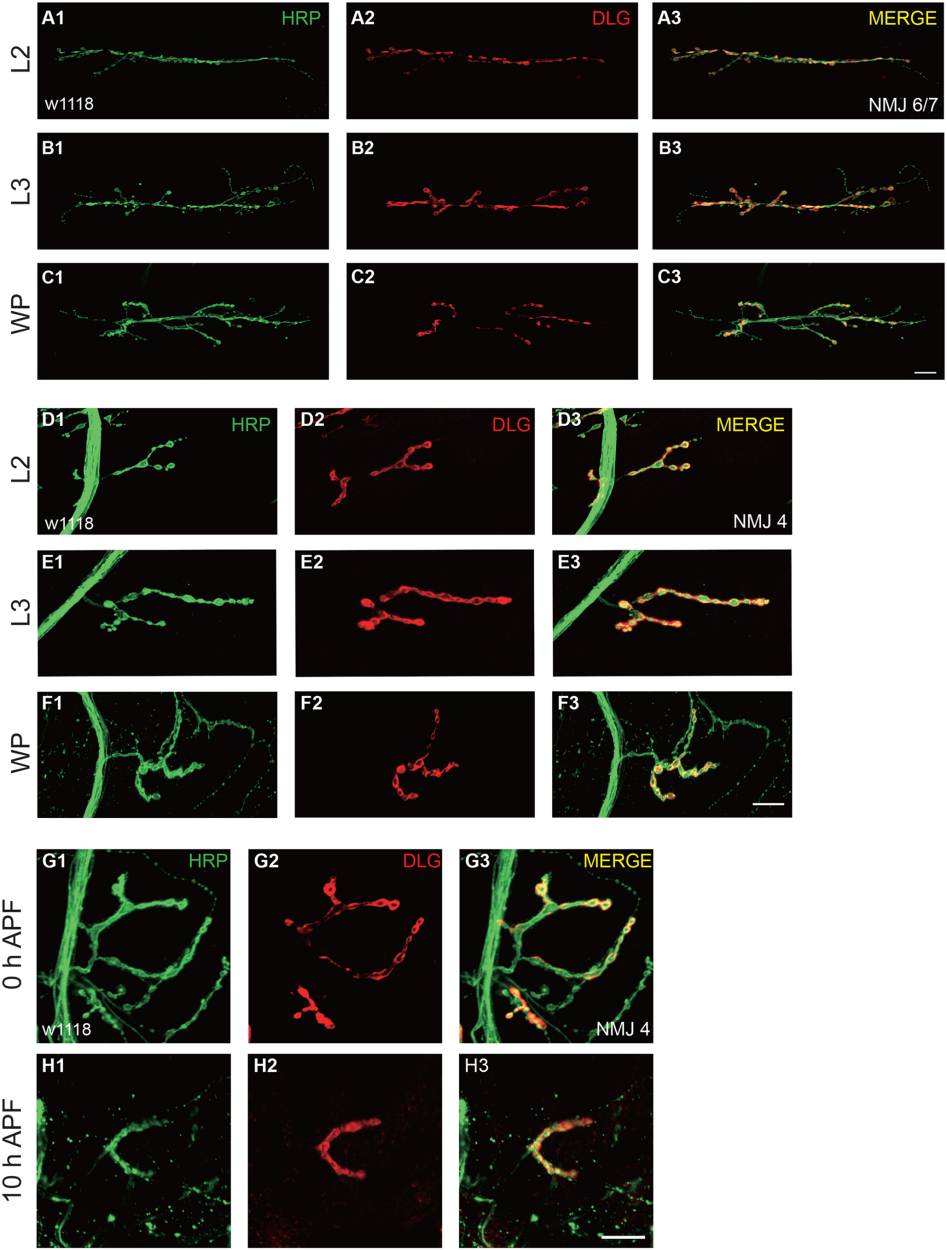
**Terminal NMJ undergo growth and dismantling during larval to pupal stages.** (A1-C3) Confocal immunofluorescence images of NMJ at muscle 6/7 of wild-type (w1118) *Drosophila* from the second instar larvae (A1-A3), the third instar larvae (B1-B3), and white prepupae (C1-C3), stained with anti-HRP (green) and anti-Dlg (red). (D1-F3) Representative confocal immunofluorescence images of NMJ at muscle 4 of wild-type (w1118) *Drosophila* from the second instar larvae (D1-D3), the third instar larvae (E1-E3), and white prepupae (F1-F3), stained with anti-HRP (green) and anti-Dlg (red). (G1-H3) Representative confocal images of NMJ at muscle 4 of wild-type (w1118) *Drosophila* at white prepupae (G1-G3) and 10 h APF (H1-H3) stages. Scale bars: 200μm (C3, F3, and H3).

### Axon bundles of motor neuron undergo degeneration during metamorphosis

Given that ecdysone signaling has extensively been proven to trigger the metamorphosis of *Drosophila* to remodel its nerve system, we wondered whether the *Drosophila* motor system would undergo remodeling after the white prepupae stages, to work in concert with MB γ neuron and ddaC sensory neurons when ecdysone signaling is activated (Lee et al., 2000). To this end, we drove mCD8-GFP by OK6-Gal4 to track the motor neurons and our confocal images showed the middle axon bundles extended from VNC underwent clearance via large-scale degeneration, axons break to form discontinuous fragments (Fig. 2A-H), rather retraction, elimination starts at a particular end and gradually shortens, in NMJ (Boulanger and Dura, 2015; Boulanger et al., 2012; Liu et al., 2010) to reconstruct adult-specific motor neural system during metamorphosis. In addition, we attempted to pin down the detailed time point when the motor axon starts to degenerate, and ultimately, we found it occurs approximately at 14 h APF that axon starts to form a discontinuous axon bundle, which should be recognized as a sign of degeneration (Fig. 2C). To further confirm it, we drove mCD8-GFP by Elav-Gal4, a pan-neuronal gal4, to track the middle axon bundles of motor neurons and our confocal images showed the similar results to OK6 (Fig. S1A-G). Moreover, to better gain insight into the different clearance mechanism between the middle axon bundles and terminal NMJ, we analyzed the developmental situations of NMJ at white prepupae and 10 h APF. In these experiments we observed the terminal NMJ started to prune its terminal boutons prior to axon bundles (Fig. 3G1-H3). Collectively, our results for the first time demonstrate that motor axon bundle of *Drosophila* prune via degeneration rather retraction.

### Motor neuron prunes almost all the proximal axon bundles at 22 h APF

Based on the results above, we next attempted to understand whether motor axon bundles were completely eliminated in line with 18 h APF in MB γ neuron (Yang et al., 2021). To this end, additional observations were needed to discern the precise time point. Importantly, it was a key goal to reveal axon pruning defects after attenuating the function of candidate genes in the future. Further time course analyses of motor axon pruning during metamorphosis illustrated that a small number of axons remained during 16-20 h APF (Fig. 2D-F) and those larval motor axons were eventually pruned away approximately at 22 h APF (Fig. 2G). To further scrutinize the remodeling process of motor axons, we assayed and achieved analogous results at 24 h APF that, in line with 22 h APF (Fig. 2H). To exclude the pruning of axon bundles is the result of mother cell losing, we conducted sets of experiments and showed the neuronal cell bodies were not being lost at 14 and 22 h APF (Fig. S2A-D). Taken together, the above results demonstrate that it occurs approximately at 22 h APF that motor neuron of *Drosophila* prune almost all their proximal axon bundles. Therefore, future studies to determine the motor axon pruning defect will focus on 22 h APF.

### Microtubule (MT) disassembly precedes to axon pruning during metamorphosis

**Fig. 4.**
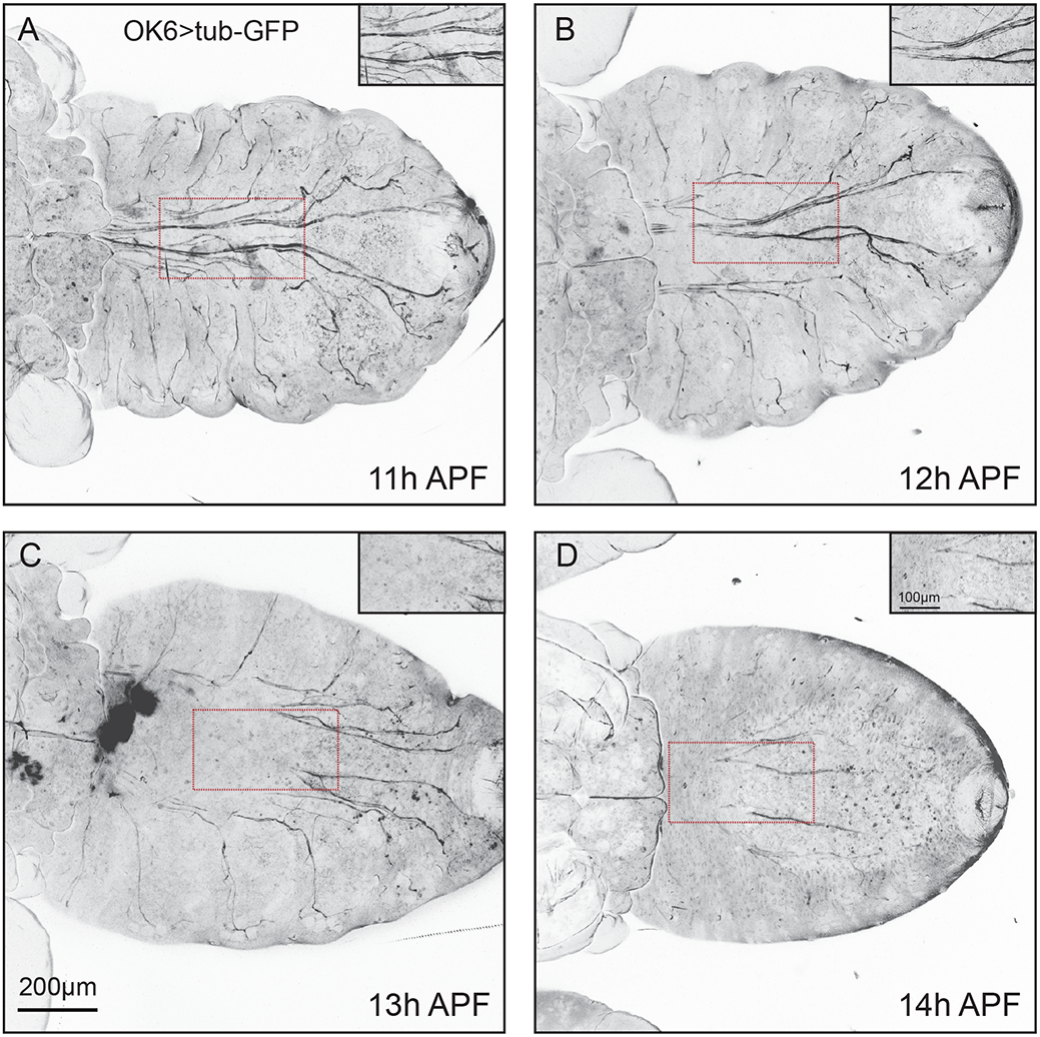
**MTs disassembly prior to axon pruning during metamorphosis.** (A-D) Representative live confocal imaging of tub-GFP-labelled MTs in motor neurons driven with OK6-Gal4 (OK6). At 11 h APF, the axonal MTs showed the sign of disassembly with a few discontinued filaments (A), at 12 h APF MTs are further destabilized and fragmented (B). The majority of MTs have disappeared at 13 h APF to 14 h APF (C,D). Scale bars: 200 μm (normal images), 100 μm (zoomed images).

It has long been thought that localized breakage of MTs is one of the earliest cellular events, contributes to neuronal pruning (Bu et al., 2021; Lee et al., 2009; Williams and Truman, 2005). In order to visualize the MTs, we drove tubulin-GFP by OK6-Gal4 to specifically label the MTs in motor neurons. Consistent with previous studies that dendrites of ddaC neuron's MTs become destabilized and fragmented during metamorphosis, we observed MTs disassembly in axon of motor neuron at a relatively earlier time compared to axon degeneration. At 11 h APF, the MTs in axon showed the sign of disassembly with the appearance of a few discontinued filaments (Fig. 4A), MTs are further destabilized and fragmented as development progress (Fig. 4B). Apparently, the majority of MTs have disappeared at 13 h APF to 14 h APF (Fig. 4C,D), whereas axon bundles in motor neuron start to degenerate at 14 h APF as our previous data suggested. Thus, the breakage of axonal MTs in motor neuron is required for initiating axon pruning.

### TGF-β signaling, ecdysone signaling, JNK signaling and ubiquitin-proteasome system are required for axon pruning of motor neuron

**Fig. 5.**
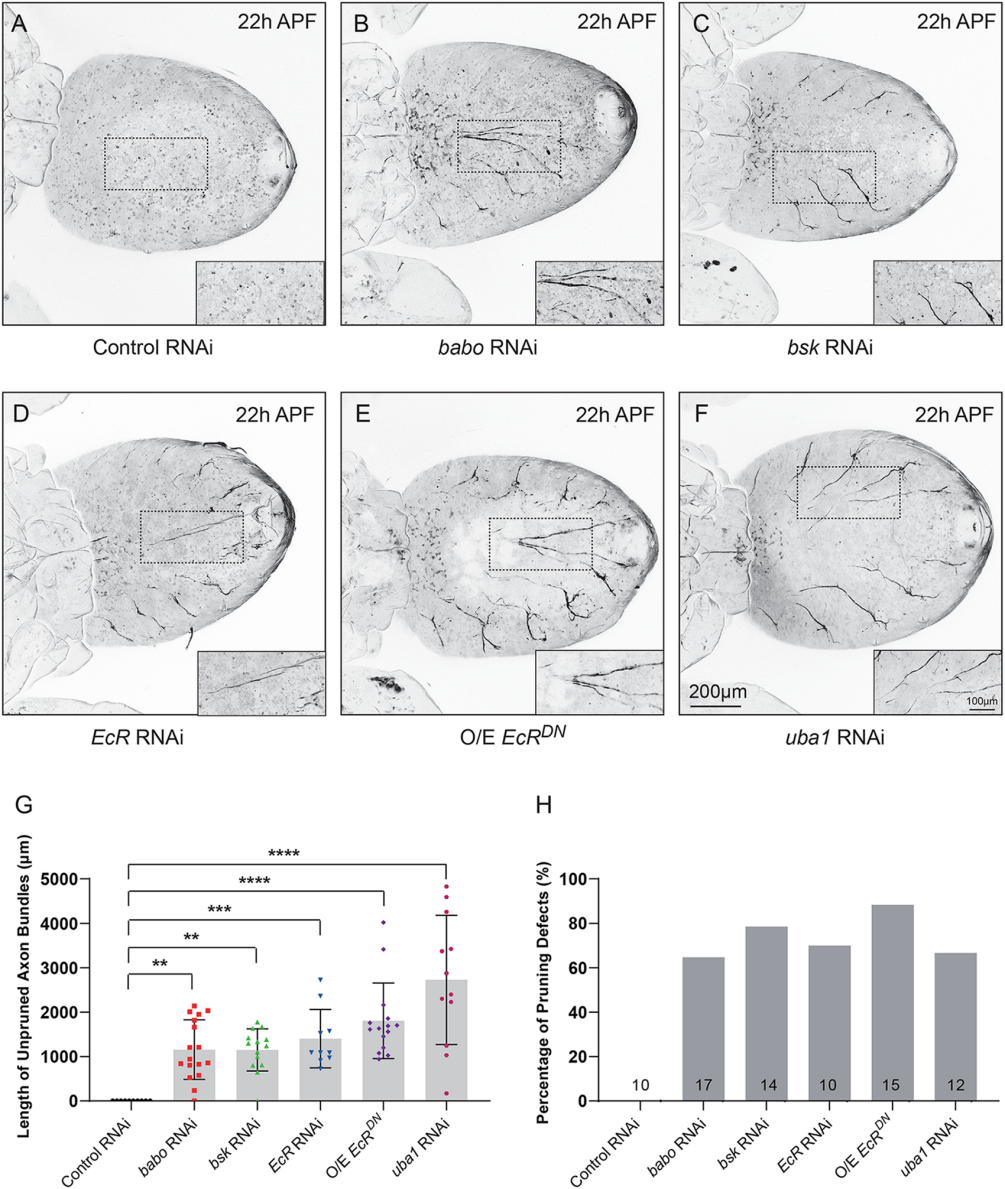
**TGF-β signaling, ecdysone signaling, JNK signaling and ubiquitin-proteasome system are required for axon pruning of motor neuron.** (A-F) Representative confocal live images of the development of mCD8-GFP-labelled motor neurons of Control RNAi, *babo* RNAi, *bsk* RNAi, *EcR* RNAi, O/E *EcR^DN^*, and *uba1* RNAi at 22 h APF. The zoomed images are found at the bottom right of each image. (G) Quantification of total length of unpruned motor axon bundles at 22 h APF. (H) Quantification of the percentage of motor axon pruning defect in control and mutant motor neurons at 22 h APF. The number of pupae (*n*) in each group is shown on the bars. O/E represents as overexpress. Data are presented as mean±s.e.m. from three independent experiments. **P<0.01; ***P<0.001; ****P<0.0001 (one-way ANOVA with Bonferroni test). Scale bars: 200 μm (normal images), 100 μm (zoomed images).

We next attempted to identify possible regulatory factors of motor axon pruning, which may act conservatively with the known regulators of axon pruning of MB γ neuron in the central nervous system. Previous studies have demonstrated that axon pruning of MB γ neuron requires the activation of signals or factors, including TGF-β signaling, ecdysone signaling, JNK signaling, ubiquitin-proteasome system and so on (Bornstein et al., 2015; Lee et al., 2000; Watts et al., 2003; Zheng et al., 2003). We next investigated whether these signaling modulate motor axon pruning during development. We therefore knocked down Baboon (Babo), a *Drosophila* TGF-β/activin type I receptor (Zheng et al., 2003); EcR-B1, the ecdysone receptor B1 isoform (Lee et al., 2000); Bsk, the *Drosophila* ortholog of the JNK (Bornstein et al., 2015); Uba1, the ubiquitin activating enzyme 1 (Kuo et al., 2005; Watts et al., 2003) and overexpressed the dominant-negative form of EcR (EcR^DN^) (Kuo et al., 2005) specific in motor neuron via a variety of available RNAi lines and dominant-negative form of transgene. The neurons expressing the control RNAi construct completely eliminated pupal motor axon at 22 h APF (*N*=10, BSC35785, Fig. 5A,G and H). In contrast, knockdown of *babo*, via RNAi transgene (*N*=17, THU5256), led to axon pruning defects in the vast majority of pupae at the same time point (64.7%, Fig. 5B,G and H). Likewise, analysis of JNK mutant neuron via *bsk* RNAi (*N*=14, BSC36643), revealed consistent axon pruning defects in 78.57% of the mutant pupae (Fig. 5C,G and H). RNAi knockdown and dominant-negative (DN) compromised the function of EcR via OK-Gal4 caused consistent prominent axon pruning defects at 22 h APF (70%, *N*=10, BSC9327, Fig. 5D,G and H; 66.67%, *N*=15, BSC9452, Fig. 5E,G and H, respectively). The expression of *uba1* RNAi transgene, via OK-Gal4, led to axon pruning defects in the vast majority of pupae (88.33%, *N*=12, THU2127, Fig. 5F,G and H). To further confirm the efficiently of RNAi and dominant-negative lines, we therefore knocked down or overexpress these regulatory genes with the utilized lines driven by PPK-Gal4 to check a known mutant phenotype of dendrite pruning defects in ddaC neuron. Compared to the controls, knockdown of babo, bsk, EcR, uba1 and overexpress EcR^DN^ consistently caused dendrite pruning defects (Fig. S3A-H). In conclusion, our studies raise the possibility that motor axon of *Drosophila* could be emerged as an excellent model to greatly facilitate the study developmental axon pruning due to the ease of live-cell imaging and the conservative regulatory mechanisms.

## DISCUSSION

A critical step in the establishment of the mature nervous system is selectively removal of superfluous or inappropriate neuronal processes without death of the parental neurons, referred to as pruning (Riccomagno and Kolodkin, 2015; Schuldiner and Yaron, 2015). Despite the considerable progress made in previous years, our understanding of the molecular mechanisms of neurite pruning is still incomplete. Previous studies reported that the *Drosophila* MB γ neuron provides a unique platform to study mechanism of axon remodeling due to its temporal and spatial stereotypy as well as the wide spectrum of genetic tools available. In this study, we identified motor neuron of *Drosophila* as an ideal model to study axon specific pruning due to the ease of live imaging that is limited in MB and the conservative regulatory mechanisms. We first show that *Drosophila* motor neuron pruned their middle axon bundles rather terminal NMJ through degeneration, it occurs approximately at 14 h APF and the axons were completely eliminated at 22 h APF. Furthermore, our data suggest that the regulatory signals of axon pruning in MB act conservatively within motor system. We thus propose that motor neuron of *Drosophila* would be emerged as a novel model system to explore cellular and molecular mechanisms of axon specific pruning.

### Live-cell imaging to monitor the development status of *Drosophila* motor neurons

*Drosophila* ddaC sensory neurons and MB γ neuron selectively prune their larval dendrites/axon to sculpt the nervous system during early metamorphosis (Yu and Schuldiner, 2014). Alternatively, dendrite pruning of ddaC neurons are usually investigated via live-cell imaging, while dissection and fixation are substantially used for assaying MB γ neuron axon pruning (Lee et al., 2000; Rui et al., 2020a). Thus, it still lacks an effective experimental method or excellent model system to analysis axon specific pruning via live-cell imaging. To directly monitor axon remodeling, we evaluated various cell types and our results finally verified that *Drosophila* motor neurons, stretching their axon bundles from soma at ventral nerve cord (VNC) to innervate muscles, are unambiguous for observing via live-cell imaging during development. Therefore, it will be interesting to study axon specific pruning with *Drosophila* motor neuron.

### *Drosophila* motor neurons eliminate their axon bundles via degeneration during metamorphosis

Whether *Drosophila* motor neuron can be used as an appropriate model to reveal the cellular and molecular mechanisms of axon pruning? Although live-cell imaging can be utilized to monitor the developmental axonal remodeling of *Drosophila* motor neuron, a physiological remodeling status is still lacking. Stereotyped refinement of axon bundle was uncovered during the development of *Drosophila*. We show in the present study that, *Drosophila* motor neuron prune their axon bundles via degeneration, similar to dendrite or axon remodeling in ddaC and MB γ neuron. Moreover, we further testify that it is approximately at 14 h APF when motor axon begins to form the discontinuous axon bundle to break the axon's integrity. Following, *Drosophila* motor neuron removes almost all the axon bundles at 22 h APF, while it is 16-18 h APF for dendrite/axon clearance of ddaC neuron and MB γ neuron. This may reflect that motor neuron axon is more stable and the regulatory mechanisms of axon pruning in motor neuron might unique during metamorphosis. Notably, 22 h APF should be a key time point that will be served to substantially screen and identify novel axon pruning governors.

### *Drosophila* motor neurons prune their axon bundles and terminal NMJ through different ways

Surprisingly, two groups have suggested that motor neuron sculpts the axonal terminal NMJ predominantly through retraction, exhibiting molecular markers of retraction as opposed to degeneration. The nuclear receptor pathway including FTZ-F1 and HR39 regulates EcR-B1 expression in muscle and initiates muscle eliminating via transcriptional regulation of postsynaptic degradation related genes. Following, a TGFβ-like ligand is supposed to be secreted by muscle and the transduced signal produced by the dismantling muscle is received by presynaptic terminal motor neuron. Ultimately, the terminal axon is trigged to retract by presynaptic TGFβ signaling and the ftz-f1/Hr39 nuclear receptor pathways (Boulanger and Dura, 2015; Boulanger et al., 2012; Liu et al., 2010). In comparison, the pruning process of the majority of axon bundles that linked to VNC is via degeneration, a process that totally different with terminal NMJ. Here we hypothesis that the motor neuron occupies a relatively large area form VNC to muscle and the intracellular and extracellular environments are not constant. The terminal part of motor neuron that contact with muscle to form pre- and postsynapses, thus the developmental remodeling regulatory processes are associated with both muscle and motor axon as mentioned above, however, the rest of motor axon bundles away form NMJ are localized at middle cavity of body, are predominantly surrounded by macrophages (macrophage-like hemocytes), and possibly glia cells. Thus, it seems plausible that the developmental pruning of motor axon bundles might be affected by intrinsic neuronal signals and extrinsic signals from glia cell that are different with terminal NMJ. Further investigations are required to distinguish these two possible mechanisms.

### Motor neuron is an attractive platform to isolate novel axon pruning regulators

Based on our new findings, it suggests that *Drosophila* motor neuron is an ideal pruning system, support for this model comes from the results of time course analyses of motor axon pruning during metamorphosis. To further testify the reliability of this model, we provided multiple lines of evidence using RNAi knockdown and dominant negative approaches, unambiguously demonstrating that TGF-β signaling, ecdysone signaling, JNK signaling and ubiquitin-proteasome system, recognized as axon pruning regulators in MB, are also required for motor axon pruning. Nevertheless, it is still urgent to explore new axon pruning regulatory signals in motor neuron. Notably, to date, little is known about the extrinsic signals including cell adhesion, secretion, and signaling pathways from surrounding glia cells, are essential for controlling neurite pruning. Future studies towards identification of the novel intrinsic and extrinsic signals that govern motor neuron axon pruning might allow us to elucidate important pathogenesis of human neuropsychiatric diseases.

## MATERIALS AND METHODS

### Fly strains

The following stocks were obtained from Tsinghua RNAi Stock Center: UAS-babo-RNAi (THU5256), UAS- uba1-RNAi (THU2127).

The following stocks were obtained from Bloomington Stock Center (BSC): w1118, ok6-gal4, elav-gal4 (c155), PPK-Gal4, mCD8-GFP, UAS-EcR-RNAi (BSC9327), UAS-EcR-B1^DN^ (BSC9452), UAS-bsk-RNAi (BSC36643), UAS-mCherry-RNAi (BSC35785).

UAS-tubulin-GFP (Dr. Xing Liang, University of Tsinghua).

### Live imaging analysis

To image *Drosophila* motor neurons at the first instar (1rd), second instar (2rd), third instar (3rd), and WP stage, animals were first washed in PBS buffer briefly, followed by immersion with 90% glycerol. For imaging motor neurons at 12-24 h APF, pupal cases were carefully removed before they were mounted with 90% glycerol.

### Immunostaining

The immunostaining procedure were described previously (Zhang et al., 2017). The primary antibodies used in this study were as follows: rabbit anti-HRP (DSHB, 1:500), mouse anti-DLG (DSHB, 1:50), Alexa Fluor 488- and Alexa Fluor 555-conjugated secondary antibodies (Invitrogen, 1:500). For immunostaining, larvae and pupae for each set of experiments were dissected simultaneously in cold PBS and fixed with 4% formaldehyde for 30 min. Mounting was performed in VECTASHIELD mounting medium, and the samples were directly visualized at room temperature using a LSM 900 (Carl Zeiss) confocal microscope for whole NMJ staining.

### Quantification of axon

Total length of unpruned axon bundles was measured in a 275 µm×275 µm region of the whole region of body wall using ImageJ. Mutant motor neurons derived from *babo* RNAi, *bak* RNAi, EcR RNAi, EcR^DN^, and *uba1* RNAi exhibited severe axon pruning defects with full penetrance at 22 h APF. On average, 1156 µm, 1151 µm, 1404 µm, 1807 µm, and 2727 µm of total axon bundles every pupa in *babo* RNAi, *bak* RNAi, EcR RNAi, EcR^DN^, and *uba1* RNAi were remained, respectively. In contrast, all the axon bundles of control neurons were pruned away at the same time point. The pruning defect was defined by the appearance of unpruned axon bundles that were more than half of the abdominal segment.

### Quantification of dendrites

Live confocal images of ddaC neurons were performed at WP and 16 h APF stages. The length of unpruned dendrites was measured in a 275 µm×275 µm region derived from the dorsal dendritic field of ddaC neurons, ranging from the abdominal segments 2-4 (Rui et al., 2020a). The pruning defect of dendrite was defined by the length of dendrite that more than 200 µm at 16 h APF.

## Statistics

For pairwise comparison, two-tailed Student's *t*-test was applied to determine statistical significance. One-way ANOVA with Bonferroni test was applied to determine significance when multiple groups were present. Error bars in all graphs represent s.e.m. Statistical significance was defined as ****P<0.0001, ***P<0.001, **P<0.01. The number of neurons (*n*) in each group is shown on the bars.

## Supplementary Material

10.1242/biolopen.059535_sup1 Supplementary information
