# Supplementary material for: Establishment of a novel axon pruning model of Drosophila motor neuron
Source: Biol Open. 2023 Jan 6;12(1):bio059535. doi: 10.1242/bio.059535 (PMC9838636; doi:10.1242/bio.059535)
Supplement: Supplementary information [file biolopen-12-059535-s1.pdf]

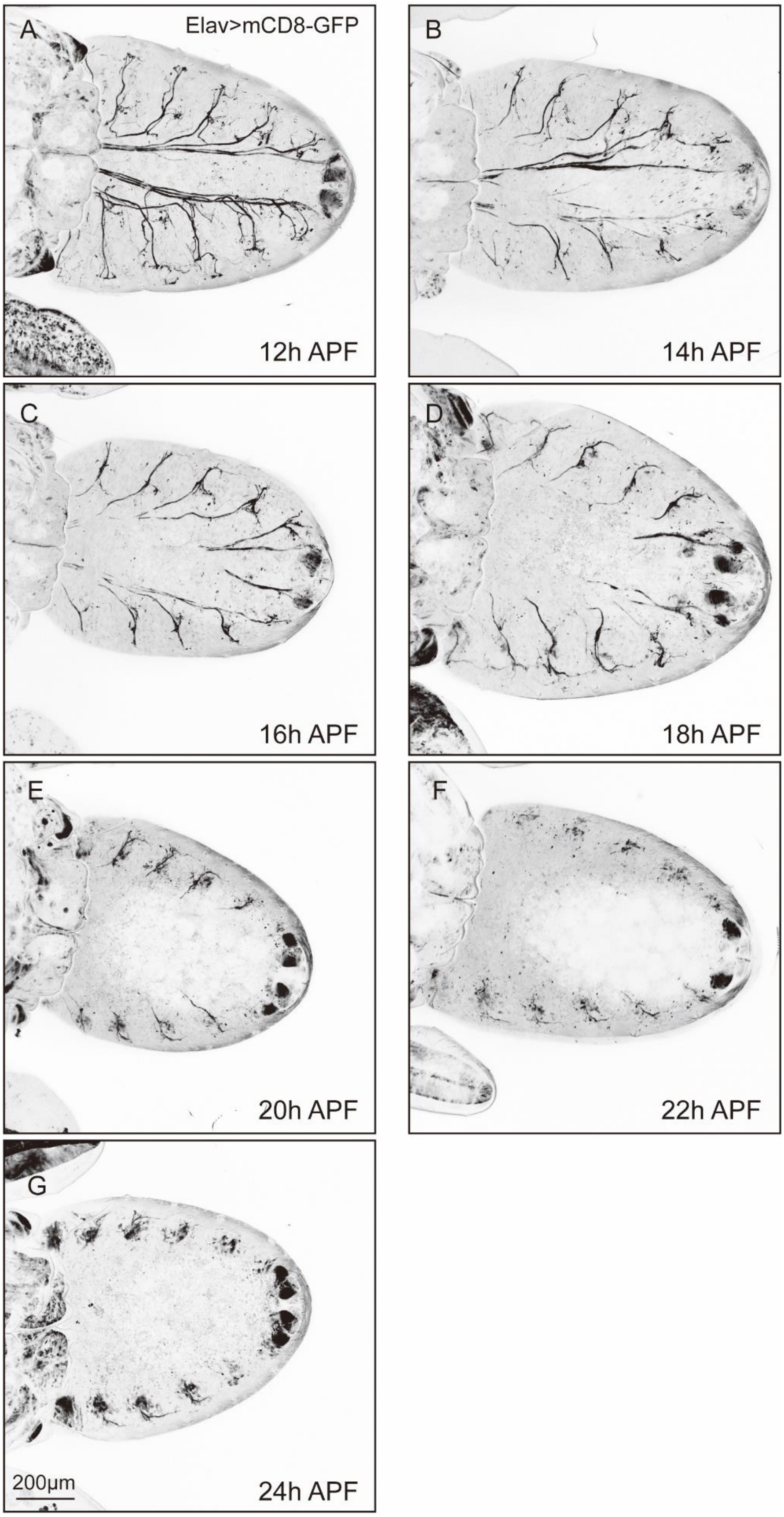

Fig. S1. Motor Neuron Pruned Its Axon Bundles During Metamorphosis.
(A-G) Confocal live imaging of the development of mCD8-GFP-labelled motor neurons driven with Elav-Gal4. At 12h APF axon bundles remain intact (A). Axon bundles appear to break at 14h APF, indicating the initiation of axonal degeneration (B). Axon bundles are further destabilized as development progress (C-G). Almost all the axon bundles are cleared at 22 h APF (F) and 24 h APF (G). Scale bars represent 200µm.

Biology Open • Supplementary information

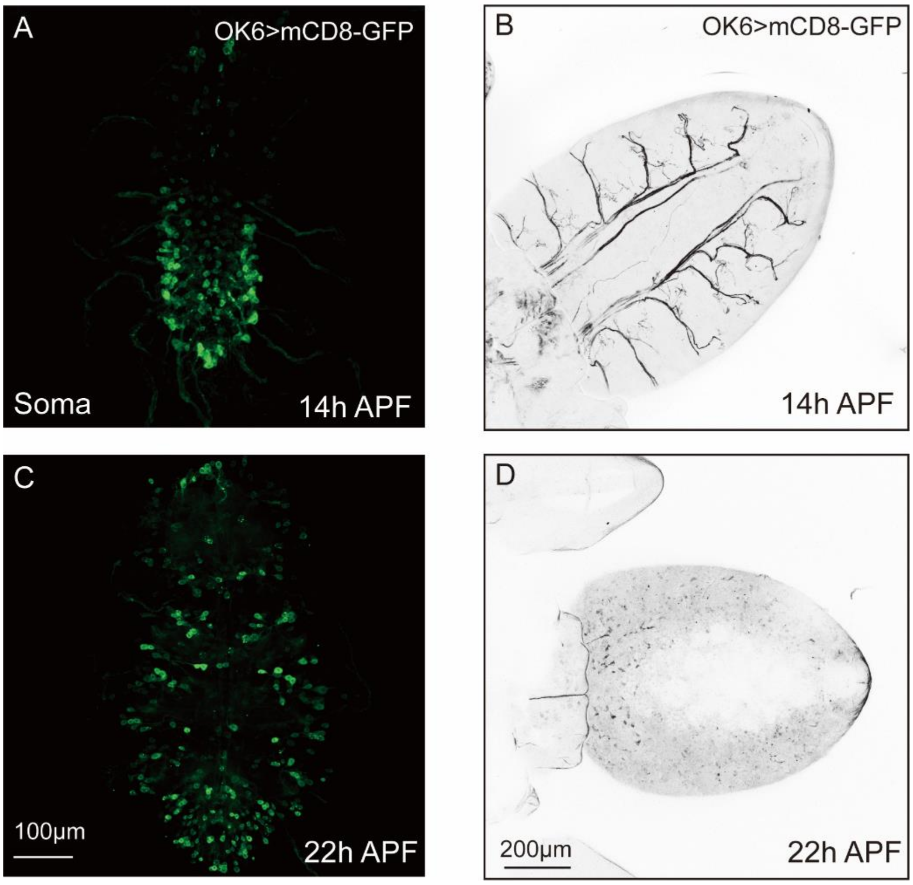

Fig. S2. Motor Neuron Pruned Its Axon Bundles without Losing Parental Neuron.
(A-D) Representative live confocal images of the development of mCD8-GFP-labelled motor neurons driven with OK6-Gal4. Somas of motor neuron in VNC at 14 h APF (A). Axon bundles at 14h APF (B). Somas of motor neuron in VNC at 22 h APF (C). Axon bundles at 22 h APF (D). Scale bars represent 100µm (A and C), 200µm (B, D).

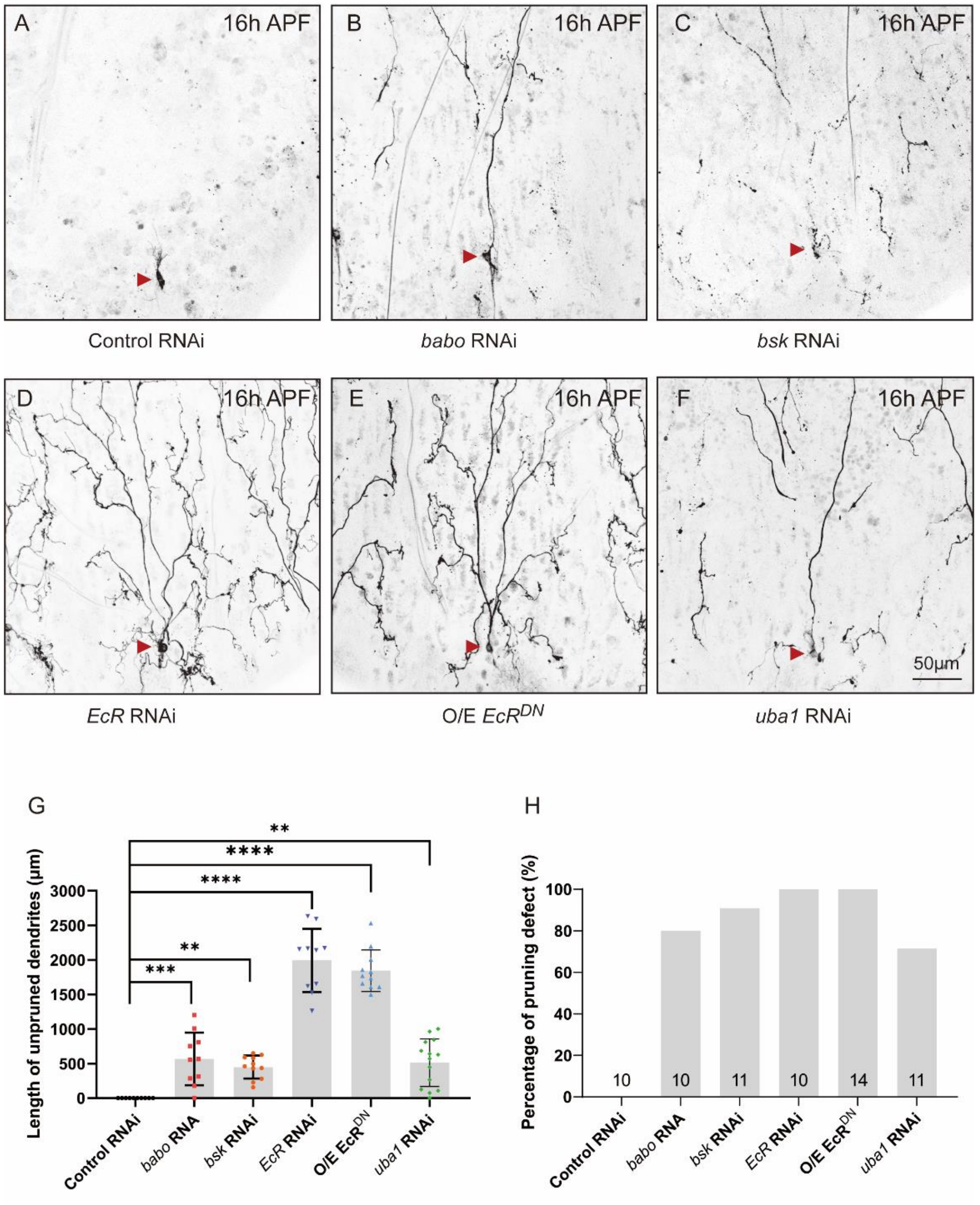

Fig. S3. Axon Pruning Defects in Utilized Lines Consistently Lead to Dendritic Pruning Defects of ddaC Neurons

(A-F) Live confocal images of ddaC neurons expressing mCD8-GFP driven by ppk-Gal4 at WP and 16 h APF. While the wild-type neurons cleared all the dendrites (A), ddaC neurons overexpressing babo RNAi (B), bak RNAi (C), EcR RNAi (D), EcRDN (E), and uba1 RNAi (F) exhibited dendrite pruning defects at 16 h APF. Red arrowheads point to the ddaC somas. (G) Quantification of total length of unpruned ddaC dendrites at 16 h APF. (H) Quantification of severing defects at 16 h APF. Scale bars represent 50 mm. O/E represents as overexpress. Data are presented as mean ± SEM. The number of samples (n) in each group is shown on the bars. **P < 0.01; ***P < 0.001; ****P < 0.0001 (one-way ANOVA with Bonferroni test). Scale bars represent 200μm.

Biology Open • Supplementary information
